# Dynamical Analysis of the Dow Jones Index Using Dimensionality Reduction and Visualization

**DOI:** 10.3390/e23050600

**Published:** 2021-05-13

**Authors:** António M. Lopes, Jóse A. Tenreiro Machado

**Affiliations:** 1 LAETA/INEGI, Faculty of Engineering, University of Porto, Rua Dr. Roberto Frias, 4200-465 Porto, Portugal; 2 Department of Electrical Engineering, Institute of Engineering, Polytechnic of Porto, Rua Dr. António Bernardino de Almeida, 431, 4249-015 Porto, Portugal; jtm@isep.ipp.pt

**Keywords:** dimensionality reduction, data visualization, clustering, time-series, complex systems

## Abstract

Time-series generated by complex systems (CS) are often characterized by phenomena such as chaoticity, fractality and memory effects, which pose difficulties in their analysis. The paper explores the dynamics of multidimensional data generated by a CS. The Dow Jones Industrial Average (DJIA) index is selected as a test-bed. The DJIA time-series is normalized and segmented into several time window vectors. These vectors are treated as objects that characterize the DJIA dynamical behavior. The objects are then compared by means of different distances to generate proper inputs to dimensionality reduction and information visualization algorithms. These computational techniques produce meaningful representations of the original dataset according to the (dis)similarities between the objects. The time is displayed as a parametric variable and the non-locality can be visualized by the corresponding evolution of points and the formation of clusters. The generated portraits reveal a complex nature, which is further analyzed in terms of the emerging patterns. The results show that the adoption of dimensionality reduction and visualization tools for processing complex data is a key modeling option with the current computational resources.

## 1. Introduction

Complex systems (CS) are composed of several autonomous entities, described by simple rules, that interact with each other and their environment. The CS give rise to a collective behavior that exhibits a much richer dynamical phenomena than the one presented by the individual elements. Often, CS exhibit evolution, adaptation, self-organization, emergence of new orders and structures, long-range correlations in the time–space domain, chaoticity, fractality, and memory effects [1,2,3,4]. The CS are not only pervasive in nature, but also in human-related activities, and include molecular dynamics, living organisms, ecosystems, celestial mechanics, financial markets, computational systems, transportation and social networks, and world and country economies, as well as many others [5,6,7,8].

Time-series analysis has been successfully adopted to study CS [9,10]. The CS outputs are measured over time and the data collected are interpreted as manifestations of the CS dynamics. Therefore, the study of the time-series allows for conclusions about the CS behavior to be reached [11,12]. Nonetheless, real-word time-series may be affected by noise, distortion and incompleteness, requiring advanced processing methods for the extraction of significant information from the data [13]. Information visualization plays a key role in time-series analysis, as it provides an insight into the data characteristics. Information visualization corresponds to the computer generation of dataset visual representations. Its main goal is to expose features hidden in the data, in order to understand the system that generated such data [14,15]. Dimensionality reduction [16] plays a key role in information visualization, since the numerical data are often multidimensional. Dimensionality reduction-based schemes try to preserve, in lower-dimensional representations, the information present in the original datasets. They include linear methods, such as classic multidimensional scaling (MDS) [17], principal component [18], canonical correlation [19], linear discriminant [20] and factor analysis [21], as well as nonlinear approaches, such as non-classic MDS, or Sammon’s projection [22], isomap [23], Laplacian eigenmap [24], diffusion map [25], t-distributed stochastic neighbor embedding (t-SNE) [26] and uniform manifold approximation and projection (UMAP) [27].

Financial time series have a complex nature and their dynamic characterization is challenging. The Dow Jones Industrial Average (DJIA) is an important financial index and is adopted in this paper as a dataset generated by a CS. The paper explores an alternative strategy to the classical time-domain analysis, by combining the concepts of distance and dimensionality reduction with computational visualization tools. The DJIA time-series of daily close values is normalized and segmented, yielding a number of objects that characterize the DJIA dynamics. These objects are vectors, whose time-length and partial time-overlap represent a compromise between time resolution and memory length. The objects are compared using various distances and their dissimilarities are used as the input to different dimensionality reduction and information visualization algorithms, namely hierarchical clustering (HC), MDS, t-SNE and UMAP. The aforementioned algorithms construct representations of the original dataset, where time is a parametric variable. The  structure of the plots is further analyzed in terms of the emerging patterns. The formation of clusters and the evolution of the patterns over time maps a dynamical behavior with discontinuities for periods where the memory is somehow lost. Numerical experiments illustrate the feasibility and effectiveness of the method for the processing of complex data.

The paper organization is summarized as follows. Section 2 reviews mathematical fundamental concepts, namely the distances and the algorithms adopted in the study for processing and visualizing data. Section 3 introduces the DJIA dataset. Section 4 analyses the data and interprets the results in the light of the distances used. Section 5 assesses the effect of the time-length and overlap of the segmenting window. Section 6 presents the conclusions.

## 2. Mathematical Concepts and Tools

### 2.1. Distances

Given two points $v_{i}$ and $v_{j}$ in a set X, the function $d\left(v_{i},v_{j}\right):X\times X\to \left[0,+\infty \right]$ represents a distance between the points if, and only if, it satisfies the conditions: identity of indiscernibles, symmetry and triangle inequality [28].

In this paper, the distances {Arccosine, Canberra, Dice, Divergence, Euclidean, Jaccard, Lorentzian, Manhattan, Sørenson, Generalized$\}=\left\{d_{1},\ldots ,d_{10}\right\}$ are considered. Therefore, given $v_{i}=\left(v_{i1},\ldots ,v_{iP}\right)$ and $v_{j}=\left(v_{j1},\ldots ,v_{jP}\right)$ in a *P*-dimensional space, P, the 10 distances are given by [28]:

(1) $$\mathrm{Arccosine}:\;\;d_{1}\left(v_{i},v_{j}\right)=\arccos\left(\frac{\sum_{k=1}^{P}v_{ik}\cdot v_{jk}}{\sqrt{\sum_{k=1}^{P}v_{ik}^{2}}\sqrt{\sum_{k=1}^{P}v_{jk}^{2}}}\right),$$

(2) $$\mathrm{Canberra}:\;\;d_{2}\left(v_{i},v_{j}\right)=\sum_{k=1}^{P}\frac{|v_{ik}-v_{jk}|}{|v_{ik}\left|+\right|v_{jk}|},$$

(3) $$\mathrm{Dice}:\;\;d_{3}\left(v_{i},v_{j}\right)=\frac{\sum_{k=1}^{P}{\left(v_{ik}-v_{jk}\right)}^{2}}{\sum_{k=1}^{P}v_{ik}^{2}+\sum_{k=1}^{P}v_{jk}^{2}},$$

(4) $$\mathrm{Divergence}:\;\;d_{4}\left(v_{i},v_{j}\right)=2\sum_{k=1}^{P}\frac{{\left(v_{ik}-v_{jk}\right)}^{2}}{{\left(v_{ik}+v_{jk}\right)}^{2}},$$

(5) $$\mathrm{Euclidean}:\;\;d_{5}\left(v_{i},v_{j}\right)=\sqrt{\sum_{k=1}^{P}{\left(v_{ik}-v_{jk}\right)}^{2}},$$

(6) $$\mathrm{Jaccard}:\;\;d_{6}\left(v_{i},v_{j}\right)=\frac{\sum_{k=1}^{P}{\left(v_{ik}-v_{jk}\right)}^{2}}{\sum_{k=1}^{P}v_{ik}^{2}+\sum_{k=1}^{P}v_{jk}^{2}-\sum_{k=1}^{P}v_{ik}v_{jk}},$$

(7) $$\mathrm{Lorentzian}:\;\;d_{7}\left(v_{i},v_{j}\right)=\sum_{k=1}^{P}\ln\left(1+|v_{ik}-v_{jk}|\right),$$

(8) $$\mathrm{Manhattan}:\;\;d_{8}\left(v_{i},v_{j}\right)=\sum_{k=1}^{P}\left|v_{ik}-v_{jk}\right|,$$

(9) $$Sø\mathrm{renson}:\;\;d_{9}\left(v_{i},v_{j}\right)=\frac{\sum_{k=1}^{P}\left|v_{ik}-v_{jk}\right|}{\sum_{k=1}^{P}|v_{ik}\left|+\right|v_{jk}|},$$

(10) $$\begin{array}{c} \mathrm{Generalized}:\;\;d_{10}\left(v_{i},v_{j}\right)=\sum_{r=1}^{9}\lambda_{r}\frac{d_{r}\left(v_{i},v_{j}\right)}{\max[d_{r}\left(v_{i},v_{j}\right)]}, \end{array}$$

where $\lambda_{r}\in R$, $\sum_{i=1}^{9}\lambda_{r}=1$.

The distances (1)–(9) have advantages and disadvantages, meaning that they unravel specific features embedded in the data, while neglecting others. Therefore, the ‘generalized’ distance $d_{10}$ may eventually capture a multi-perspective information by combining (1)–(9) in a complementary form.

Other techniques [29] and distances [28] can also be adopted to compare the data. However, a more extensive overview and utilization of a larger number of alternatives is out of the scope of the paper.

### 2.2. Dimensionality Reduction and Visualization

In the next subsections, the dimensionality reduction and visualization techniques that are adopted for data processing are presented, namely the HC, MDS, t-SNE and UMAP.

Given a set of *N* objects, $v_{i}$, i=1,…,N, in space P, all methods require the definition of a distance $d(v_{i},v_{j})$, i,j=1,…,N, between the objects *i* and *j*.

#### 2.2.1. The Hierarchical Clustering

The HC groups similar objects and represents them in a 2-dim locus. The algorithm involves two main steps [30]. In the first, the HC constructs a matrix of distances, $D=[d\left(v_{i},v_{j}\right)]$, of dimension N×N, where $d\left(v_{i},v_{j}\right)=d\left(v_{j},v_{i}\right)$. In the second step, the algorithm arranges the objects in a hierarchical structure and depicts them in a graphical portrait, namely, a hierarchical tree or a dendrogram. This is achieved by means of two alternative techniques: the divisive and the agglomerative procedures. In the divisive scheme, all objects start in one single cluster and end in separate clusters. This is done by iteratively removing the ‘outsiders’ from the least cohesive cluster. In the agglomerative scheme, each object starts in its own cluster and all end in one single cluster. This is accomplished by successive iterations that join the most similar clusters. The HC requires the specification of a linkage criterion for measuring the dissimilarity between clusters. Often, the average-linkage, $d_{av}\left(R,S\right)$, is adopted [31], where *R* and *S* represent two clusters. Therefore, denoting $d\left(v_{R},v_{S}\right)$ the distance between a pair of objects $v_{R}\in R$ and $v_{S}\in S$, in the clusters *R* and *S*, respectively, we have:

(11) $$d_{av}\left(R,S\right)=\frac{1}{∥R∥∥S∥}\sum_{v_{R}\in R,v_{S}\in S}d\left(v_{R},v_{S}\right).$$

The reliability of the clustering can be assessed by the cophenetic coefficient cc [32]

(12) $$cc=\frac{\sum_{i<j}\left[d\left(v_{i},v_{j}\right)-\mathrm{av}\left(d(v_{i},v_{j})\right)\right]\left[\hat{d}\left(t_{i},t_{j}\right)-\mathrm{av}\left(\hat{d}\left(t_{i},t_{j}\right)\right)\right]}{\sqrt{\left[\sum_{i<j}{\left[d\left(v_{i},v_{j}\right)-\mathrm{av}\left(d(v_{i},v_{j})\right)\right]}^{2}\right]\left[\sum_{i<j}{\left[\hat{d}\left(t_{i},t_{j}\right)-\mathrm{av}\left(\hat{d}\left(t_{i},t_{j}\right)\right)\right]}^{2}\right]}},$$

where $\left\{v_{i},v_{j}\right\}$ and $\left\{t_{i},t_{j}\right\}$ stand for the original objects and their HC representations, respectively, av(·) denotes the average of the input argument, and  $\hat{d}\left(t_{i},t_{j}\right)$ represents the cophenetic distance between $t_{i}$ and $t_{j}$. We always obtain 0≤cc≤1, with the limits corresponding to bad and good clustering, respectively. Additionally, the original and the cophenetic distances can be represented in a scatter plot denoted by Shepard diagram. A good clustering corresponds to points located close to a 45${}^{\circ }$ line.

#### 2.2.2. The Multidimensional Scaling

The MDS includes a class of methods that represent high-dimensional data in a lower dimensional space, while preserving the inter-point distances as much as possible. The matrix $D=[d\left(v_{i},v_{j}\right)]$ feeds the MDS dimensionality reduction and visualization algorithm. The MDS tries to find the positions of *Q*-dimensional objects, $t_{i}$, with i=1,…,N, represented by points in space Q, so that Q≤P, while producing a matrix $T=[\hat{d}\left(t_{i},t_{j}\right)]$ that approximates D. This is accomplished by means of an optimization procedure that tries to minimize a fitness function. Usually, the stress cost function, S, is adopted

(13) $$S={\left[\sum_{i<j}{\left[d\left(v_{i},v_{j}\right)-\hat{d}\left(t_{i},t_{j}\right)\right]}^{2}\right]}^{\frac{1}{2}}.$$

The Sammon criterion is an alternative, yielding

(14) $$S={\left[\frac{\sum_{i<j}{\left[d\left(v_{i},v_{j}\right)-\hat{d}\left(t_{i},t_{j}\right)\right]}^{2}}{\sum_{i<j}{\left[d(v_{i},v_{j})\right]}^{2}}\right]}^{\frac{1}{2}}.$$

The ‘quality’ of the MDS is assessed by comparing the original and the reproduced information. This can be accomplished by means of the Shepard diagram, which depicts $d(v_{i},v_{j})$ versus $\hat{d}\left(t_{i},t_{j}\right)$. Additionally, since the stress *S* decreases monotonically with the dimension *Q*, the user can establish a compromise between the two variables. Often, the practical choice is Q=2 or Q=3, since those values yield a direct graphical representation in the embedding space. Nevertheless, if the MDS locus is unclear, then the user must adopt another measure $d(v_{i},v_{j})$ until a suitable representation is obtained.

#### 2.2.3. The t-Distributed Stochastic Neighbor Embedding

The t-SNE [26] is a technique for visualizing high-dimensional datasets, with  applications including computer security [33], music analysis [34], bioinformatics [35] and other areas [36,37].

The algorithm comprises two main stages. In the first, for each pair of objects ($v_{i},v_{j}$), i,j=1,…,N, the t-SNE constructs a joint probability distribution $p_{ij}$ measuring the similarity between $v_{i}$ and $v_{j}$, in such a way that similar (dissimilar) objects are assigned a higher (lower) probability

(15) $$p_{ij}=\frac{p_{j|i}+p_{i|j}}{2N},$$

(16) $$p_{j|i}=\left\{\begin{array}{cc} \frac{\exp[-d{\left(v_{i},v_{j}\right)}^{2}/\left(2\sigma_{i}^{2}\right)]}{\sum_{k\neq i}\exp\left[-d{\left(v_{i},v_{k}\right)}^{2}/\left(2\sigma_{i}^{2}\right)\right]}, & j\neq i\\ 0, & j=i \end{array}\right.,$$

where $p_{ij}=p_{ji}$, $p_{ii}=0$, $\sum_{i,j}p_{ij}=1$ and $\sum_{j}p_{j|i}=1,\forall i,j$. The parameter $\sigma_{i}^{2}$ represents the variance of the Gaussian kernel that is centered on $v_{i}$. A particular value of $\sigma_{i}$ induces a probability distribution $P_{i}$, over all of the other datapoints. In other words, $P_{i}$ represents the conditional probability distribution over all other datapoints given the datapoint $v_{i}$. The t-SNE searches for the value of $\sigma_{i}$ that generates a distribution $P_{i}$ with the value of perplexity specified by

(17) $$\mathrm{perplexity}\left(P_{i}\right)=2^{H(P_{i})},$$

where $H(P_{i})$ is the Shannon entropy of $P_{i}$

(18) $$H\left(P_{i}\right)=\sum_{j}p_{j|i}\log_{2}\left(p_{j|i}\right).$$

As a result, the variation in the Gaussian kernel is adapted to the density of the data, meaning that smaller (larger) values of $\sigma_{i}$ are used in denser (sparser) parts of the data space. The perplexity can be interpreted as a smooth measure of the effective number of $v_{i}$ neighbors. Typical values of $\mathrm{perplexity}(P_{i})$ are in the interval [5,50].

In the second stage, the t-SNE calculates the similarities between pairs of points in Q

(19) $$q_{ij}=\frac{q_{j|i}+q_{i|j}}{2N},$$

(20) $$q_{ij}=\left\{\begin{array}{cc} \frac{\left(1+|\right|t_{i}-t_{j}{\left|\right|}^{2}{)}^{-1}}{\sum_{k\neq l}\left(1+|\right|t_{k}-t_{l}{\left|\right|}^{2}{)}^{-1}}, & j\neq i\\ 0, & j=i \end{array}\right.,$$

where the symbol ||·|| denotes the 2-norm of the argument, $q_{ij}=q_{ji}$, $q_{ii}=0$, $\sum_{i,j}q_{ij}=1$ and $\sum_{j}q_{j|i}=1,\forall i,j$.

The t-SNE performs an optimization, while attempting to minimize the Kullback–Leibler (KL) divergence between the Gaussian distribution of the points in space P and the Student *t*-distribution of the points in the embedding space Q:

(21) $$KL=\sum_{i\neq j}p_{ij}\ln\frac{p_{ij}}{q_{ij}}.$$

The minimization scheme starts with a given initial set of points in Q, and the algorithm uses the gradient descent

(22) $$\frac{\partial KL}{\partial t_{i}}=4\sum_{j}\left(p_{ij}-q_{ij}\right)\left(t_{i}-t_{j}\right)\left(1+|\right|t_{i}-t_{j}{\left|\right|}^{2}{)}^{-1}.$$

The KL divergence between the modeled input and output distributions is often used as a measure of the quality of the results.

#### 2.2.4. The Uniform Manifold Approximation and Projection

The UMAP is a recent technique [27] for clustering and visualizing high-dimensional datasets, which seeks to accurately represent both the local and global structures embedded in the data [38,39].

Given a distance, $d(v_{i},v_{j})$, between pairs of objects $v_{i}$ and $v_{j}$, i,j=1,…,N, and the number of neighbors to consider, *k*, the UMAP starts by computing the *k*-nearest neighbors of $v_{i}$, $N_{i}$, with respect to $d(v_{i},v_{j})$. Then, the algorithm calculates the parameters $\rho_{i}$ and $\sigma_{i}$, for each datapoint $v_{i}$. The parameter $\rho_{i}$ represents a nonzero distance from $v_{i}$ to its nearest neighbor and is given by

(23) $$\rho_{i}=\min_{j\in N_{i}}\left\{d\left(v_{i},v_{j}\right)|d\left(v_{i},v_{j}\right)>0\right\}.$$

The parameter $\rho_{i}$ is important to ensure the local connectivity of the manifold. This means that it yields a locally adaptive exponential kernel for each point.

The constant $\sigma_{i}$ must satisfy the condition

(24) $$\log_{2}k=\sum_{j\in N_{i}}\exp\left[\frac{-\max(0,d\left(v_{i},v_{j}\right)-\rho_{i})}{\sigma_{i}}\right],$$

determined using binary search.

The algorithm constructs a joint probability distribution $p_{ij}$ measuring the similarity between $v_{i}$ and $v_{j}$, in such a way that similar (dissimilar) objects are assigned a higher (lower) probability

(25) $$p_{ij}=p_{j|i}+p_{i|j}-p_{j|i}p_{i|j},$$

(26) $$p_{j|i}=\left\{\begin{array}{cc} \exp\left[\frac{-\max(0,d\left(v_{i},v_{j}\right)-\rho_{i})}{\sigma_{i}}\right], & j\neq i\\ 0, & j=i \end{array}\right.,$$

where $p_{ij}=p_{ji}$, $p_{ii}=0$, $\sum_{i,j}p_{ij}=1$ and $\sum_{j}p_{j|i}=1,\forall i,j$.

In the second stage, the UMAP computes the similarities between each pair of points in the space Q

(27) $$q_{ij}=q_{j|i}+q_{i|j}-q_{j|i}q_{i|j},$$

(28) $$q_{ij}=\left\{\begin{array}{cc} {\left[1+a||t_{i}-t_{j}{\left|\right|}^{2b}\right]}^{-1}, & j\neq i\\ 0, & j=i \end{array}\right.,$$

where $q_{ij}=q_{ji}$, $q_{ii}=0$, $\sum_{i,j}q_{ij}=1$ and $\sum_{j}q_{j|i}=1,\forall i,j$. The constants a,b∈R are either user-defined or are determined by the algorithm given the desired separation between close points, $\delta \in R^{+}$, in the embedding space Q

(29) $${\left[1+a||t_{i}-t_{j}{\left|\right|}^{2b}\right]}^{-1}\approx \left\{\begin{array}{cc} 1, & t_{i}-t_{j}\leq \delta \\ \exp[-\left(t_{i}-t_{j}\right)-\delta ], & t_{i}-t_{j}>\delta \end{array}\right..$$

The UMAP performs an optimization while attempting to minimize the cross-entropy CE between the distribution of points in P and Q

(30) $$CE=\sum_{i\neq j}\left[p_{ij}\ln\frac{p_{ij}}{q_{ij}}-\left(1-p_{ij}\right)\ln\frac{1-p_{ij}}{1-q_{ij}}\right].$$

The minimization scheme starts with a given initial set of points in Q. The UMAP uses the Graph Laplacian to assign initial low-dimensional coordinates, and then proceeds with the optimization using the gradient descent

(31) $$\frac{\partial CE}{\partial t_{i}}=\sum_{j}\left[\frac{2ab{\left[d\left(t_{i},t_{j}\right)\right]}^{2(b-1)}}{1+a{\left[d\left(t_{i},t_{j}\right)\right]}^{2b}}p_{ij}-\frac{2b}{{\left[d\left(t_{i},t_{j}\right)\right]}^{2}\left(1+a{\left[d\left(t_{i},t_{j}\right)\right]}^{2b}\right)}\left(1-p_{ij}\right)\right]\left(t_{i}-t_{j}\right).$$

## 3. Description of the Dataset

The prototype dataset representative of a given CS corresponds to the DJIA daily closing values from 28 December 1959 up to 12 March 2021. Each week includes 5 working days. Occasional missing data are obtained by means of linear interpolation. The resulting time series $x=\{x_{k}:k=1,\ldots ,L\}$ comprises L=15970 values, $x_{k}$, covering approximately half a century.

Often, we pre-process x in order to reduce the sensitivity to a high variation in the numerical values, yielding $\tilde{x}=\left\{\Phi_{q}\left(x_{k}\right):k=1,\ldots ,L\right\}$. Functions $\Phi_{q}\left(\cdot \right)$, which are commonly adopted, are the logarithm of the values, the logarithm of the returns and the normalization by the arithmetic mean, av(x), and the standard deviation, σ(x), given by

(32) $$\Phi_{1}\left(x_{k}\right)=\ln x_{k},$$

(33) $$\Phi_{2}\left(x_{k}\right)=\left\{\begin{array}{cc} \ln\frac{x_{k+1}}{x_{k}}, & k=1,\ldots ,L-1\\ 0, & k=L \end{array}\right.,$$

(34) $$\Phi_{3}\left(x_{k}\right)=\frac{x_{k}-\mathrm{av}\left(x\right)}{\sigma (x)}.$$

**Figure 1:**
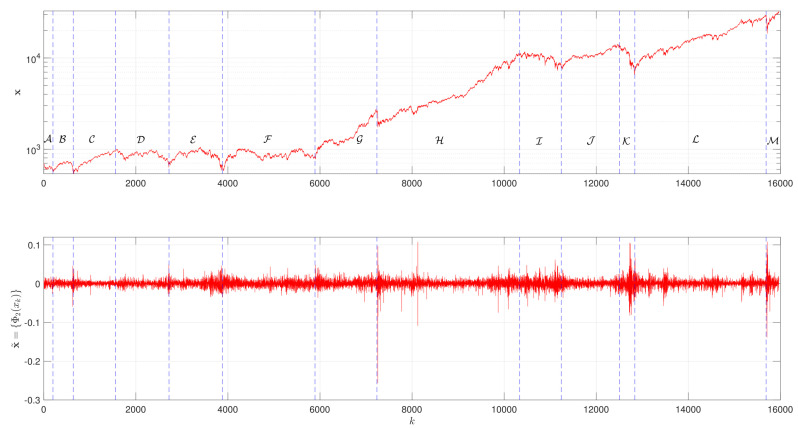
The evolution of the time-series x and $\tilde{x}=\left\{\Phi_{2}\left(x_{k}\right):k=1,\ldots ,L\right\}$, in the period from 28 December 1959 up to 12 March 2021.

Figure 1 depicts the evolution of x, as well as the logarithm of the returns $\tilde{x}\;=\;\left\{\Phi_{2}\left(x_{k}\right):k=1,\ldots ,L\right\}$, which reveals a fractal nature. We verify the existence of 13 main periods denoted from A to M. For k∈[1,640], corresponding to the periods A and B, the values of $x_{k}$ are small, starting with a decrease, followed by a recovering trend. This behavior is followed by a sustainable increase in the DJIA during k∈[640,1555], period C. The interval k∈[1555,5890] corresponds to the periods D, E and F, which are characterized by an overall stagnation in the between of severe crises. For k∈[5890,7237], that is, period G, we have an important rising trend, interrupted abruptly, but rapidly recovered, marking the beginning of period H for k∈[7237, 10,340]. For k∈[10,340, 11,240], corresponding to period I, the DJIA reveals a decreasing trend. This behavior is followed by the period J, during the interval k∈[11,240, 12,500], characterized by a sustained increase in the DJIA values. For k∈[12,500, 12,840], the period K reveals a strong falling trend. Then, recovery initiates and a rising trend is verified during the period L, that is, for k∈[12,840, 15,690]. This period is interrupted suddenly, but rapidly, recovered, signaling the beginning of M, corresponding to k∈[15,690, 15,970]. Table 1 summarizes the DJIA main periods and some historical events occurred during 28 December 1959 up to 12 March 2021.

To assess the dynamics of the DJIA, the time-series $\tilde{x}$ is segmented into $N=1+\frac{L-W}{(1-\alpha )W}$, where *W* is the window length, α∈[0,1] stands for the window overlapping factor, and · denotes the floor function. Therefore, the *i*th, i=1,…,N, window consists of the vector $v_{i}=\left\{\Phi_{q}\left(x_{p}\right):p=\left(i-1\right)\left(1-\alpha \right)W+1,\ldots ,\left(i-1\right)\left(1-\alpha \right)W+W\right\}$.

**Figure 2:**
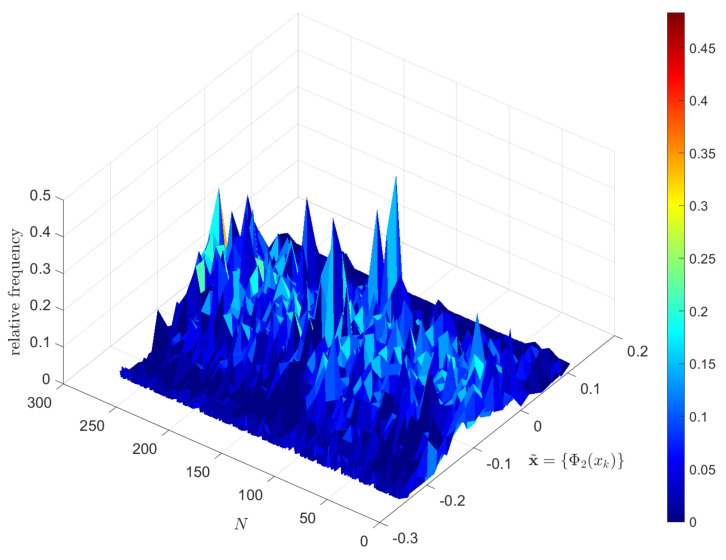
The histogram of $\tilde{x}=\left\{\Phi_{2}\left(x_{k}\right):k=1,\ldots ,L\right\}$ for consecutive disjoint time windows (α=0) and W=60.

Figure 2 portraits the histogram of $\tilde{x}=\left\{\Phi_{2}\left(x_{k}\right):k=1,\ldots ,L\right\}$ for consecutive disjoint windows (α=0) and W=60. We verify the existence of fat tails in the statistical distribution, as well as a ‘noisy’ behavior, which are also verified for other functions $\Phi_{q}$ and values of α and *W*.

## 4. Analysis and Visualization of the DJIA

The DJIA time-series x is normalized using expression (34), yielding $\tilde{x}=\left\{\Phi_{3}\left(x_{k}\right):k=1,\ldots ,L\right\}$. Naturally, other types of pre-processing are possible, but the linear transform (34) is common in signal processing [40] and several experiments showed that it yields good results.

In the next subsections, $\tilde{x}$ is segmented using consecutive disjoint (α=0) time windows of length W=60 days, which yield N=266 objects, $v_{i}$, with i=1,…,N. These objects are processed by the dimensionality reduction and visualization methods, while adopting different distances (1)–(9) to quantify the dissimilarities between objects. For the generalized distance $d_{10}$, given by expression (10), since no a priori preference for a given formula is set, we adopt identical weights, that is, $\lambda_{r}=\frac{1}{9}$, r=1,…,9. The values of α and *W* were chosen experimentally. Obviously, other values could have been adopted, but those used lead to a good compromise between time resolution and suitable visualization.

### 4.1. The HC Analysis and Visualization of the DJIA

**Figure 3:**
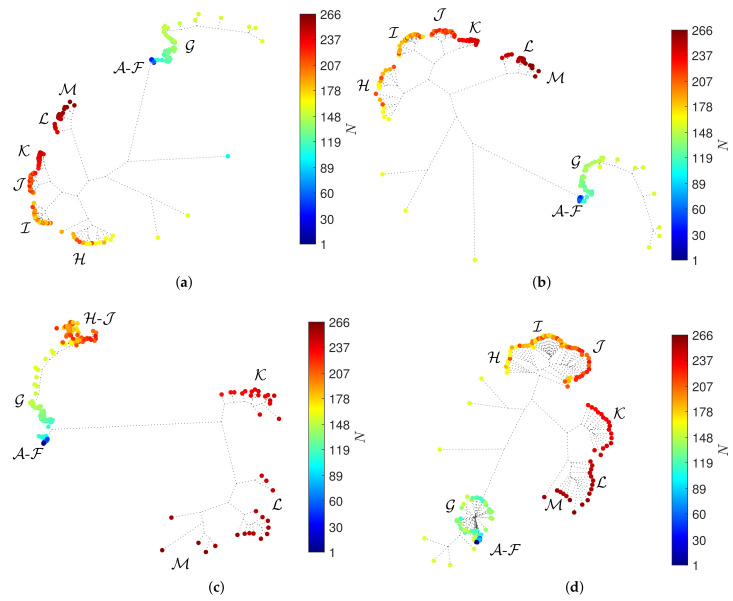
The hierarchical trees obtained by the HC for α=0 and W=60 (N=266) with four distances: (**a**) $d_{2}$; (**b**) $d_{3}$; (**c**) $d_{5}$; (**d**) $d_{10}$. The circular marks correspond to objects (window vectors) and the colormap represents the arrow of time.

The neighbor-joining method [41] and the successive (agglomerative) clustering using average-linkage are adopted, as implemented by the software Phylip [42] with the option neighbor. Figure 3 depicts the HC trees with the distances $d_{2}$, $d_{3}$, $d_{5}$ and $d_{10}$. The circular marks correspond to objects (window vectors) and the colormap represents the arrow of time. We verify that the HC has difficulty in separating the periods A-F and, for distance $d_{5}$, this difficulty is also observed for the periods H-J. For other distances, we obtain loci of the same type.

The HC loci reflect the relationships between objects, but the interpretation of such loci is difficult due to the presence of many objects and because we are constrained to 2-dim visual representations. The reliability of the clustering, that is, how well the hierarchical trees reproduce the original dissimilarities of the original objects in the dataset, was verified. Nevertheless, we do not include the Shepard diagrams for the sake of parsimony.

### 4.2. The MDS Analysis and Visualization of the DJIA

**Figure 4:**
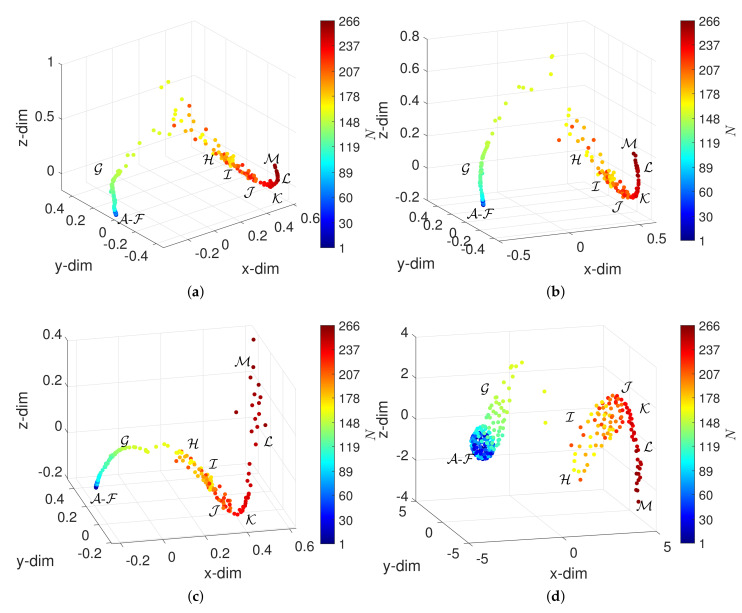
The 3-dim loci obtained by the MDS for α=0 and W=60 (N=266) with four distances: (**a**) $d_{2}$; (**b**) $d_{3}$; (**c**) $d_{5}$; (**d**) $d_{10}$. The circular marks correspond to objects (window vectors) and the colormap represents the arrow of time.

We now visualize the DJIA behavior using the MDS. The Matlab function mdscale with the Sammon nonlinear mapping criterion is adopted. Figure 4 depicts the 3-dim loci obtained for α=0 and W=60 (N=266) with the distances $d_{2}$, $d_{3}$, $d_{5}$ and $d_{10}$.

The reliability of the 3-dim loci was verified through the standard Shepard and stress plots, which showed that the objects in the embedding space Q reproduce those in the original space P. Those diagrams are not depicted for the sake of parsimony. We verify that the MDS unravels patterns compatible with the DJIA 13 periods A-M. However, the algorithm cannot discriminate between them. The patterns are composed by two ‘segments’ formed by objects that reveal an almost continuous and smooth evolution in time. Each segment translates into a DJIA dynamics exhibiting strong memory effects that are captured by the visualization technique with the adopted distance. The transition between segments corresponds to some discontinuity where the memory of past values is somehow lost.

**Figure 5:**
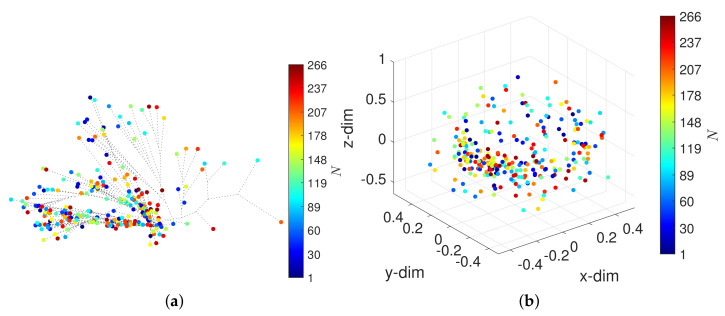
The loci obtained for α=0 and W=60 (N=266) with the correlation distance $d_{11}$: (**a**) hierarchical tree; (**b**) MDS locus.

For other distances, we obtain loci of several types. However, it should be noted that often the definition of an adequate distance (in the sense of assessing the dynamical effects) necessitates some numerical trials. Different distances can lead to valid visual representations, but may be unable to capture the features of interest. For example, the correlation distance, $d_{11}$, given by

(35) $$\mathrm{correlation}:d_{12}\left(v_{i},v_{j}\right)={\left(1-\frac{\sum_{k=1}^{P}\left[v_{ik}-\mathrm{av}\left(v_{i}\right)\right]\left[v_{jk}-\mathrm{av}\left(v_{j}\right)\right]}{\sqrt{\sum_{k=1}^{P}{\left[v_{ik}-\mathrm{av}\left(v_{i}\right)\right]}^{2}}\sqrt{\sum_{k=1}^{P}{\left[v_{jk}-\mathrm{av}\left(v_{j}\right)\right]}^{2}}}\right)}^{\frac{1}{2}},$$

leads to the loci shown in Figure 5, revealing that neither the HC nor the MDS can capture the memory effects embedded in the dataset.

### 4.3. The t-SNE Analysis and Visualization of the DJIA

**Figure 6:**
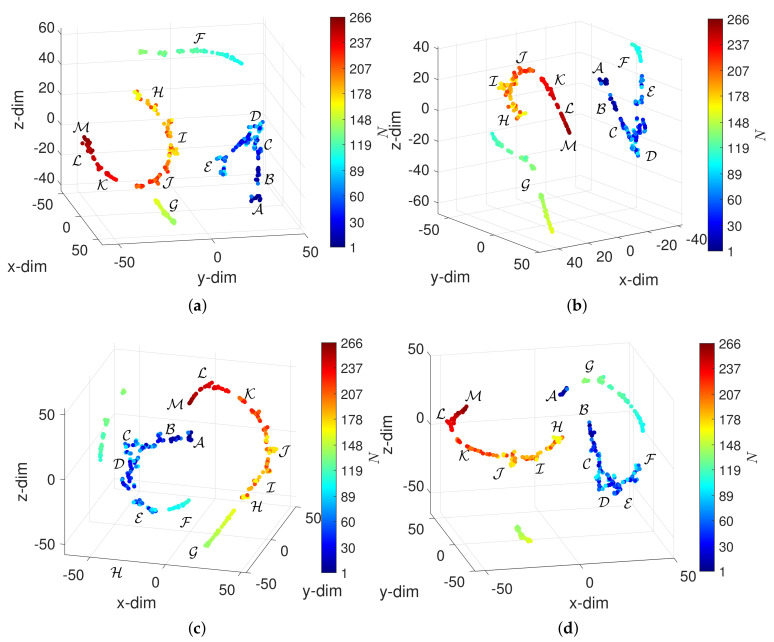
The 3-dim maps obtained by the t-SNE for α=0 and W=60 (N=266) with four distances: (**a**) $d_{2}$; (**b**) $d_{3}$; (**c**) $d_{5}$; (**d**) $d_{10}$. The circular marks correspond to objects (window vectors) and the colormap represents the arrow of time.

The Matlab function tsne was adopted to visualize the dataset $\tilde{x}=\left\{\Phi_{3}\left(x_{k}\right):k=1,\ldots ,L\right\}$. The algorithm was set to exact and the value 5 was given to the Exaggeration and the Perplexity. These values were adjusted by trial in order to obtain good visualization. The Exaggeration corresponds to the size of natural clusters in data. A large exaggeration creates relatively more space between clusters in the embedding space Q. The Perplexity is related to the number of local neighbors of each point. All other parameters kept their default values. Figure 6 depicts the 3-dim loci obtained for the distances $d_{2}$, $d_{3}$, $d_{5}$ and $d_{10}$. The loci reveal that the t-SNE can arrange objects according to their the periods A-M and that the plots generated with the different distances are similar.

### 4.4. The UMAP Analysis and Visualization of the DJIA

**Figure 7:**
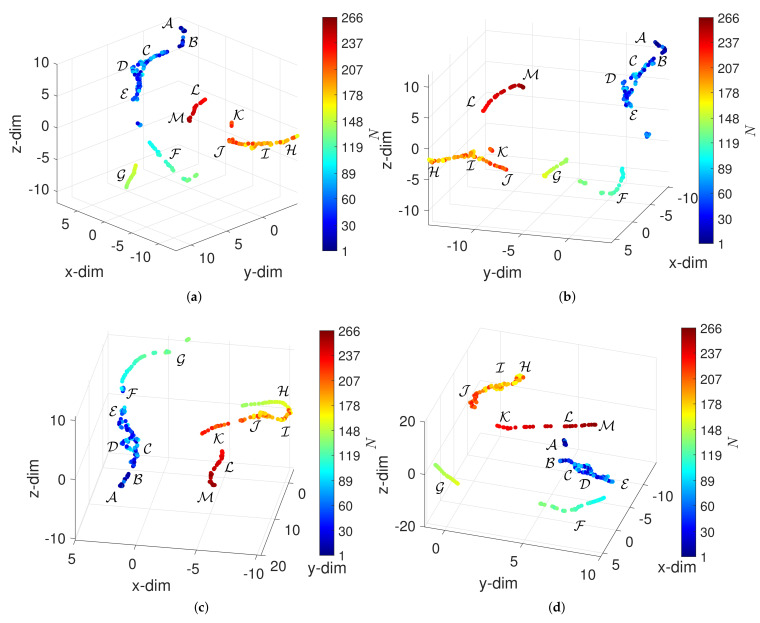
The 3-dim loci obtained by the UMAP for α=0 and W=60 (N=266) with four distances: (**a**) $d_{2}$; (**b**) $d_{3}$; (**c**) $d_{5}$; (**d**) $d_{10}$. The circular marks correspond to objects (window vectors) and the colormap represents the arrow of time.

For implementing the UMAP dimensionality reduction and visualization, we adopted the Matlab UMAP code, version 2.1.3, developed by Stephen Meehan et al. [43]. The function run_umap was used with parameters n_neighbors and min_dist set to 5 and 0.2, respectively, adjusted by trial and error in order to obtain good visualization. These parameters correspond directly to *k* and δ introduced in Section 2.2.4. All other parameters are set to their default values. Figure 7 depicts the 3-dim loci obtained for the distances $d_{2}$, $d_{3}$, $d_{5}$ and $d_{10}$.

The UMAP can organize objects in Q according to their characteristics, identifying well the periods A-M, independently of the adopted distance. Therefore, we conclude that both the t-SNE and the UMAP perform better than the MDS in representing the DJIA dynamics. The visualization has only slight variations with the distance adopted to compare objects.

## 5. Assessing the Effect of W and α in the Visualization of the DJIA Dynamics

The window width and overlap, *W* and α, represent a compromise between time resolution and memory length. In this section, we study the effect of these parameters on the patterns generated by the HC, MDS, t-SNE and UMAP. The analysis was performed for all distances and several combinations of *W* and α. The results are presented for the Canberra distance, $d_{2}$, and the cases summarized in Table 2, where W={90,60,30,10} and α={0,0.2,0.5}. For other distances, we obtain similar conclusions.

**Figure 8:**
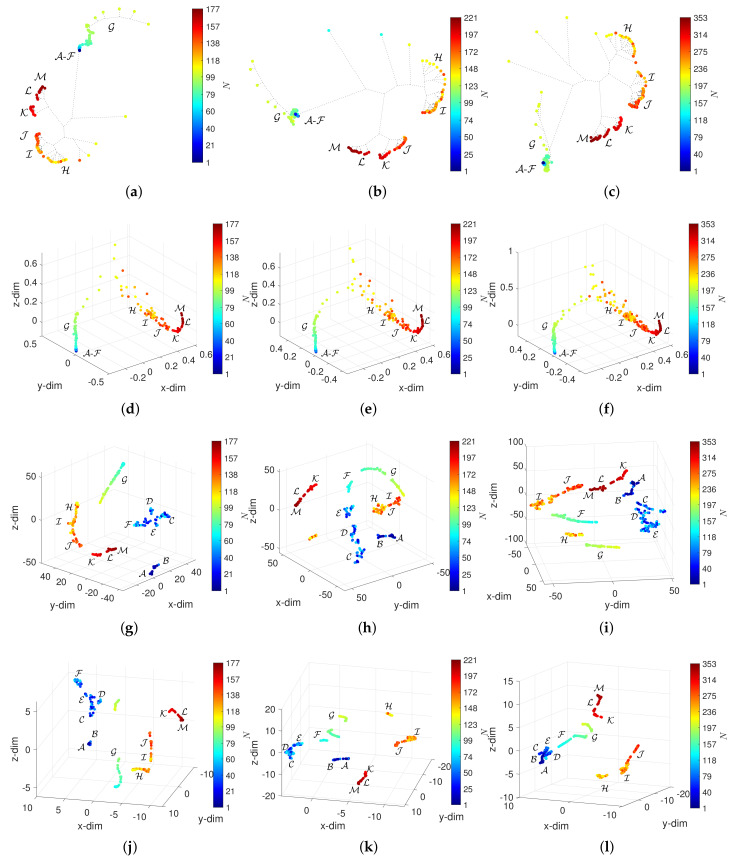
The 3-dim loci obtained for $d_{2}$ and W=90: (**a**) HC and α=0 ($E_{1}$); (**b**) HC and α=0.2 ($E_{2}$); (**c**) HC and α=0.5 ($E_{3}$); (**d**) MDS and α=0 ($E_{1}$); (**e**) MDS and α=0.2 ($E_{2}$); (**f**) MDS and α=0.5 ($E_{3}$); (**g**) t-SNE and α=0 ($E_{1}$); (**h**) t-SNE and α=0.2 ($E_{2}$); (**i**) t-SNE and α=0.5 ($E_{3}$); (**j**) UMAP and α=0 ($E_{1}$); (**k**) UMAP and α=0.2 ($E_{2}$); (**l**) UMAP and α=0.5 ($E_{3}$).

**Figure 9:**
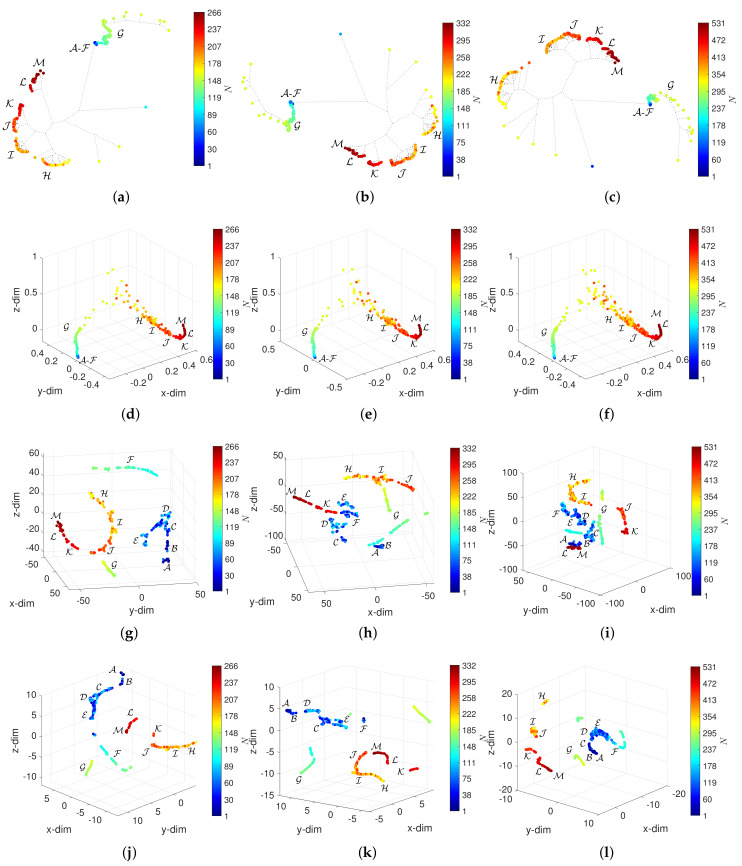
The 3-dim loci obtained for $d_{2}$ and W=60: (**a**) HC and α=0 ($E_{4}$); (**b**) HC and α=0.2 ($E_{5}$); (**c**) HC and α=0.5 ($E_{6}$); (**d**) MDS and α=0 ($E_{4}$); (**e**) MDS and α=0.2 ($E_{5}$); (**f**) MDS and α=0.5 ($E_{6}$); (**g**) t-SNE and α=0 ($E_{4}$); (**h**) t-SNE and α=0.2 ($E_{5}$); (**i**) t-SNE and α=0.5 ($E_{6}$); (**j**) UMAP and α=0 ($E_{4}$); (**k**) UMAP and α=0.2 ($E_{5}$); (**l**) UMAP and α=0.5 ($E_{6}$).

**Figure 10:**
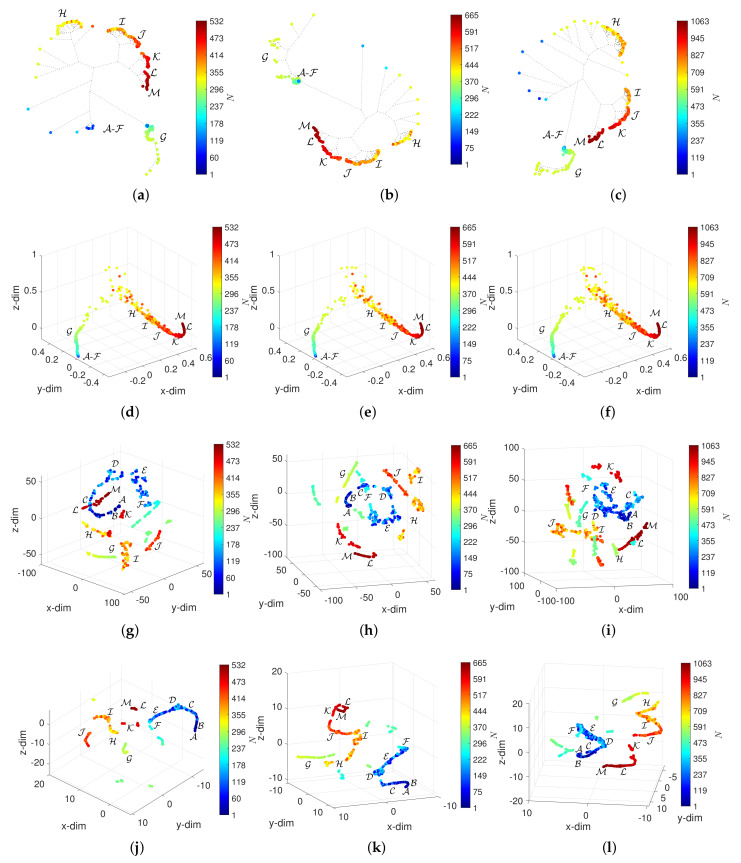
The 3-dim loci obtained for $d_{2}$ and W=30: (**a**) HC and α=0 ($E_{7}$); (**b**) HC and α=0.2 ($E_{8}$); (**c**) HC and α=0.5 ($E_{9}$); (**d**) MDS and α=0 ($E_{7}$); (**e**) MDS and α=0.2 ($E_{8}$); (**f**) MDS and α=0.5 ($E_{9}$); (**g**) t-SNE and α=0 ($E_{7}$); (**h**) t-SNE and α=0.2 ($E_{8}$); (**i**) t-SNE and α=0.5 ($E_{9}$); (**j**) UMAP and α=0 ($E_{7}$); (**k**) UMAP and α=0.2 ($E_{8}$); (**l**) UMAP and α=0.5 ($E_{9}$).

**Figure 11:**
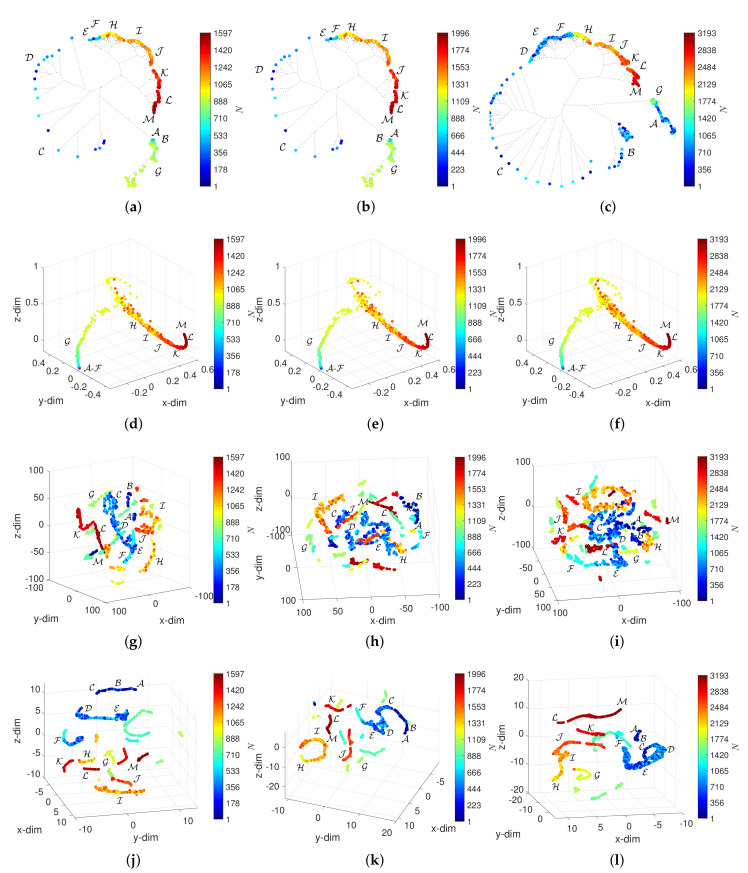
The 3-dim loci obtained for $d_{2}$ and W=10: (**a**) HC and α=0 ($E_{10}$); (**b**) HC and α=0.2 ($E_{11}$); (**c**) HC and α=0.5 ($E_{12}$); (**d**) MDS and α=0 ($E_{10}$); (**e**) MDS and α=0.2 ($E_{11}$); (**f**) MDS and α=0.5 ($E_{12}$); (**g**) t-SNE and α=0 ($E_{10}$); (**h**) t-SNE and α=0.2 ($E_{11}$); (**i**) t-SNE and α=0.5 ($E_{12}$); (**j**) UMAP and α=0 ($E_{10}$); (**k**) UMAP and α=0.2 ($E_{11}$); (**l**) UMAP and α=0.5 ($E_{12}$).

Figure 8, Figure 9, Figure 10 and Figure 11 depict the loci generated. Regarding the HC, we verify that the loci are quite insensitive to the parameter *W*, with the exception of W=10. For this value of window length, the HC can discriminate objects in the periods A-F, despite the fact that capability depends on the overlap α. For W=10 and α=0.5, the objects in A-F spread out in space, but their clusters are still unclear. Concerning the MDS, besides the density of objects, which, naturally, varies with *N*, the 3-dim loci are almost invariant with respect to the parameters *W* and α. The t-SNE and UMAP reveal a superior ability to generate patterns that correspond to dissimilarities between objects and, therefore, are able to identify the 13 periods A-M. However, for the t-SNE, this ability is weakened as the number of objects increases, *N*, meaning small values of *W* and high values of α. For such cases, the generated loci are difficult to interpret. The UMAP reveals the 13 periods A-M for all combinations of *W* and α. Moreover, for small values of *W* several sub-periods are unraveled, which directly relate to the time evolution of the DJIA.

## 6. Conclusions

This paper explored a strategy representing an alternative to the classical time analysis in the study multidimensional data generated by CS. The DJIA index of daily closing values from 28 December 1959 up to 12 March 2021 was adopted for the numerical experiments. In the proposed scheme, the original time-series was normalized and segmented, yielding a number of objects. These objects are vectors, whose dimension and overlap represent a compromise between time resolution and memory length. The objects were compared using various distances and their dissimilarities are used as the input to the four dimensionality reduction and information visualization algorithms, namely, HC, MDS, t-SNE and UMAP. These algorithms construct representations of the original dataset, where time is a parametric variable, with no a priori requirements. The algorithms are based on the minimization of the difference between the original and approximated data. The plots were analyzed in terms of the emerging patterns. Those graphical representations are composed of a number of ‘segments’, formed by objects with an almost continuous evolution in time, interlaid, eventually, by some discontinuities. This translates into the DJIA dynamics that depicts phases with visible correlation. Consequently, memory effects and transitions corresponding to some discontinuities where the memory of past values is not present. Numerical experiments illustrated the feasibility and effectiveness of the method for processing complex data. The approach can be easily extended to deal with more features and richer descriptions of the data involving a higher number of dimensions.

## Figures and Tables

**Table 1:** The DJIA main periods and some historical events occurred during 28 December 1959 up to 12 March 2021.

Period	Interval, *k*	Start Date	End Date	Main Events
A	[1,200]	28 December 1959	30 September 1960	1961 Berlin Wall; Bay of Pigs
B	[200,640]	30 September 1960	8 June 1962	
C	[640,1555]	8 June 1962	10 December 1965	1962 Cuban Missile Crisis; 1963 John F. Kennedy Assassination; 1964 Vietnam War Begins; 1965 The Great Inflation Begins
D	[1555,2720]	10 December 1965	29 May 1970	1967 The Six Day War
E	[2720,3878]	29 May 1970	6 November 1974	1972 Watergate; Munich Olympics Massacre; 1973 U.S. Involvement in Vietnam Ends; Arab Oil Embargo; 1974 President Nixon Resigns
F	[3878,5890]	6 November 1974	23 July 1982	1977 Panama Canal Treaty; 1979 Iran Hostage Crisis; 1980 Iraq - Iran War; 1981 President Reagan Shot; 1982 Falkland Islands War
G	[5890,7237]	23 July 1982	22 September 1987	1983 Grenada Invasion; 1986 U.S. Attacks Libya; Chernobyl Accident; 1987 Financial Panic; Stock Market Crash
H	[7237, 10,340]	22 September 1987	13 August 1999	1989 U.S. Invades Panama; German Unification; 1991 The Golf War; Soviet Union Collapse; 1992 Civil War in Bosnia; 1993 World Trade Center Terrorist Attack; 1995 Oklahoma Terrorist Attack; 1997 Asian Currency Crisis; Global Stock Market Rout
I	[10,340, 11,240]	13 August 1999	24 January 2003	2000 Bush - Gore Election Crisis; 2001 Terrorist Attack on World Trade Center & Pentagon; Enron Crisis; 2003 War in Iraq
J	[11,240, 12,500]	24 January 2003	23 November 2007	2004 Global War on Terror; 2005 Record High Oil Prices; 2007 Subprime Mortgage; Credit Debacle
K	[12,500, 12,840]	23 November 2007	13 March 2009	2008 Credit Crisis; Financial institution Failures
L	[12,840, 15,690]	13 March 2009	14 February 2020	2010 European Union Crisis; Massive Debt; 2011 U.S. Credit Downgrade; 2012 European Debt; 2013 U.S. Government Shutdown; 2014 Oil Price Decline; 2015 Refugee Crisis; 2016 Brexit Referendum; 2017 Trump Administration; 2018 Warnings About Climate Change; U.S. - China Trade War; President Trump Impeachment Process
M	[15,690, 15,970]	14 February 2020	12 March 2021	2020 COVID19 Pandemics; Black Lives Matter

**Table 2:** List of experiments varying *W* and α.

	*W*	α	*N*		*W*	α	*N*
$E_{1}$	90	0	177	$E_{7}$	30	0	532
$E_{2}$	90	0.2	221	$E_{8}$	30	0.2	665
$E_{3}$	90	0.5	353	$E_{9}$	30	0.5	1063
$E_{4}$	60	0	266	$E_{10}$	10	0	1597
$E_{5}$	60	0.2	332	$E_{11}$	10	0.2	1996
$E_{6}$	60	0.5	531	$E_{12}$	10	0.5	3193

## Data Availability

Not applicable.
